# Study on the removal characteristics and degradation pathways of highly toxic and refractory organic pollutants in real pharmaceutical factory wastewater treated by a pilot-scale integrated process

**DOI:** 10.3389/fmicb.2023.1128233

**Published:** 2023-03-09

**Authors:** Wei Dai, Ji-Wei Pang, Jie Ding, Yu-Qian Wang, Lu-Yan Zhang, Nan-Qi Ren, Shan-Shan Yang

**Affiliations:** ^1^State Key Laboratory of Urban Water Resource and Environment, School of Environment, Harbin Institute of Technology, Harbin, China; ^2^China Energy Conservation and Environmental Protection Group, CECEP Talroad Technology Co., Ltd., Beijing, China; ^3^National Engineering Research Center for Bioenergy, Harbin Institute of Technology, Harbin, China; ^4^School of Environmental Science and Engineering, Yancheng Institute of Technology, Yancheng, China

**Keywords:** pharmaceutical wastewater, continuous stirred tank reactor, microbial electrolysis cells, expanded sludge bed reactor, moving bed biofilm reactor, toxic pollutants

## Abstract

**Introduction:**

Pharmaceutical wastewater frequently contains high levels of toxic pollutants. If they are discharged untreated, they pose a threat to the environment. The traditional activated sludge process and the advanced oxidation process do not sufficiently remove toxic and conventional pollutants from pharmaceutical wastewater treatment plants (PWWTPs).

**Methods:**

We designed a pilot-scale reaction system to reduce toxic organic pollutants and conventional pollutants from pharmaceutical wastewater during the biochemical reaction stage. This system included a continuous stirred tank reactor (CSTR), microbial electrolysis cells (MECs), an expanded sludge bed reactor (EGSB), and a moving bed biofilm reactor (MBBR). We used this system to further investigate the benzothiazole degradation pathway.

**Results and discussion:**

The system effectively degraded the toxic pollutants (benzothiazole, pyridine, indole, and quinoline) and the conventional chemicals (COD, NH_4_^+^-N, TN). During the stable operation of the pilot-scale plant, the total removal rates of benzothiazole, indole, pyridine, and quinoline were 97.66, 94.13, 79.69, and 81.34%, respectively. The CSTR and MECs contributed the most to the removal of toxic pollutants, while the EGSB and MBBR contributed less to the removal of the four toxic pollutants. Benzothiazoles can be degraded *via* two pathways: the benzene ring-opening reaction and the heterocyclic ring-opening reaction. The heterocyclic ring-opening reaction was more important in degrading the benzothiazoles in this study.

**Conclusion:**

This study provides feasible design alternatives for PWWTPs to remove both toxic and conventional pollutants at the same time.

## 1. Introduction

Pharmaceutical compounds are one of the most concerning man-made pollutants, contaminating ever more aquatic environments around the world (Fatta-Kassinos et al., 2011; Desbiolles et al., 2018; Liu et al., 2022). Globally, between 100,000 and 200,000 tons of antibiotics alone are consumed per year. Most of these antibiotics enter the environment as starting materials or their active metabolites (Xu et al., 2007). Pharmaceutical industrial wastewater contains a significant amount of toxic, dangerous, and refractory drug compounds. These dark-colored toxic compounds usually contain a complex mixture of organic pollutants and are often very saline (Hu et al., 2017; Moradi et al., 2020; Tardy et al., 2021). This toxic pharmaceutical wastewater may harm the environment if it is released without being treated. Conventional activated sludge treatment and the advanced oxidation method used by pharmaceutical wastewater treatment plants (PWWTPs) can often not efficiently remove this complex mixture of organic and conventional pollutants from the wastewater (Rosman et al., 2018; Hu et al., 2019; Gonzaga et al., 2022; He et al., 2022; Li et al., 2022; Guo et al., 2023). Traditional anaerobic sludge systems are not very efficient in removing toxic organic pollutants, which in turn lowers the efficiency of the biochemical treatment of conventional pollutants.

Different techniques have been used to reduce the toxicity of pharmaceutical wastewater and increase its biodegradability (Chen et al., 2008; Shi et al., 2017; Yi et al., 2017; Liu et al., 2020; Asensio et al., 2021; Oberoi et al., 2022). The hydrolytic acidification process has been widely used in practical engineering because it can balance water quality, generate less sludge, and improve the biodegradability of pharmaceutical wastewater. Hydrolytic acidification has many advantages, including easily controllable conditions, simple operation, and certain tolerance to toxic substances (Guo et al., 2020; Lv et al., 2021; Zhang et al., 2021). It is, however, difficult to completely degrade pharmaceutical organic toxicants by hydrolysis acidification alone, because of its long hydraulic retention time, extensive coverage, and poor economics (Min et al., 2020). The continuous stirred tank reactor (CSTR) is commonly used during the hydrolytic-acidification process. Bioelectrochemical systems (BES), in contrast to the hydrolysis acidification process, use microorganisms to catalyze redox reactions on the electrode surface. BES can effectively treat refractory chemicals in antibiotic wastewater because of the combined actions of anodic oxidation and cathodic reduction (Liu et al., 2020; Hartl et al., 2021; Gonzaga et al., 2022). Based on whether electricity is generated during the treatment process, BES can be divided into microbial fuel cells (MFCs) and microbial electrolysis cells (MECs) (Werner et al., 2015). MECs can more effectively remove refractory organic pollutants than MFCs due to co-metabolism and electrocatalysis. Microbes cannot directly use the organic matter in pharmaceutical wastewater, and to improve the effectiveness of MECs in treating refractory pollutants during nitrification and denitrification (Liu et al., 2017), the wastewater must be modified through hydrolysis and fermentation. The pharmaceutical wastewater must be pre-treated by a combination of the hydrolysis-acidification process and BES. This pre-treatment improves the biodegradability of the wastewater and total nitrogen degradation efficiency to significantly reduce the level of organic pollutants. This is an efficient and economical pretreatment technology that is used in the treatment process of pharmaceutical wastewater.

Anaerobic granular sludge and aerobic biofilm processes are more stable than the traditional activated sludge process for the biological removal of pharmaceutical wastewater because of the impact load and toxicity of pharmaceutical wastewater (Polesel et al., 2017; Yi et al., 2017; Hu et al., 2019; Granatto et al., 2021; Wang et al., 2022). The expanded granular sludge blanket (EGSB), which is a relatively mature anaerobic reactor, is often used to remove organics from pharmaceutical wastewater. Its high-volume loading, high height-to-diameter ratio, simple operation, low energy consumption, good water stability, strong resistance to impact load (Granatto et al., 2021), and the fact that it has no moving parts are advantageous. The moving bed biofilm reactor (MBBR), which combines the advantages of the traditional activated sludge process, fluidized bed, and biological contact oxidation processes, is a new efficient pharmaceutical wastewater treatment method (Wu et al., 2023). MBBR uses the entire reactor space and combines the complementary advantages of the organisms in both the attached phase and the suspended phase to form activated sludge in the suspension growth and biofilm in the attachment growth through aeration and water flow in the aeration tank (Shi et al., 2014; Wang et al., 2020). By combining EGSB and MBBR, a significant reduction of conventional pollutants, toxic and harmful substances, biological inhibitors (including a certain concentration of antibiotics), and refractory substances in pharmaceutical wastewater, can be achieved.

This study designed a pilot-scale wastewater treatment system consisting of a CSTR + MECs + EGSB + MBBR unit to investigate its effectiveness in treating pharmaceutical wastewater. According to the author’s literature research, there is no case of combining the four systems and using them in a pilot-scale study of real pharmaceutical factory wastewater. We tested the removal of antibiotics and other refractory toxic and harmful substances and conventional indicators such as chemical oxygen demand (COD), ammonia nitrogen (NH_4_^+^-N), and total nitrogen (TN). By pretreating the pharmaceutical wastewater with hydrolysis acidification and MECs, the biodegradability of the wastewater can be enhanced, and the toxic, harmful, and bio-inhibitory refractory wastewater can be more efficiently treated. Therefore, high efficiency reactors EGSB and MBBR can be used for subsequent treatment. The EGSB and MBBR biological treatment technologies can significantly remove carbon and nitrogen from the wastewater and further reduce organic pollutants. This study provides a new way to solve the problems of high energy consumption and low efficiency in the treatment of highly toxic pharmaceutical wastewater. It has important scientific significance and engineering application value and will exert a great influence on the design and production operations of pharmaceutical wastewater treatment systems.

## 2. Materials and methods

### 2.1. Wastewater quality and quantity

The study site was a pharmaceutical wastewater factory in northeast China. The factory primarily produced more than 30 active pharmaceutical ingredients and 18 dosage forms, which included penicillin and antibiotics in the cephalosporin class in powder, capsule, and tablet form. Table 1 shows the water quality parameters from the wastewater of this pharmaceutical plant.

**Table 1:** Water quality parameters of pharmaceutical factory wastewater (unit: mg/L).

pH	NH_4_^+^-N	COD	BOD_5_	TN	TOC	UV_254_	SS	SO_4_^2−^
6.68	140.55	3,731–4,223	550–600	189.22	1757.75	≥3.0	664–958	1,098

The pharmaceutical factory wastewater had a BOD_5_/COD ratio of 13–16%. This complex wastewater contained high concentrations of toxic and harmful substances, biological inhibitors (including antibiotics), and other refractory substances. The pharmaceutical factory wastewater also contained high salinity, including chloride ion concentrations of about 3,500 mg/L and sulfate concentrations of about 1,098 mg/L. In addition, the pharmaceutical wastewater also contained a few metal ions, among which the concentrations of Cr, Fe, Cu, and Zn are 0.042, 11.60, 5.37, and 0.23 mg/L, respectively. The colored odorous wastewater foamed easily and had a lot of suspended matter. The biochemical treatment of pharmaceutical wastewater is typically challenging due to its biological toxicity. Based on gas chromatograph–mass spectrometry (GC–MS), the wastewater contains more than 100 distinct kinds of organic pollutants. The most prevalent pollutants included esters, phenols, anilines, indoles, and benzothiazoles.

### 2.2. Pilot scale experiment of PWWTP

**Figure 1:**
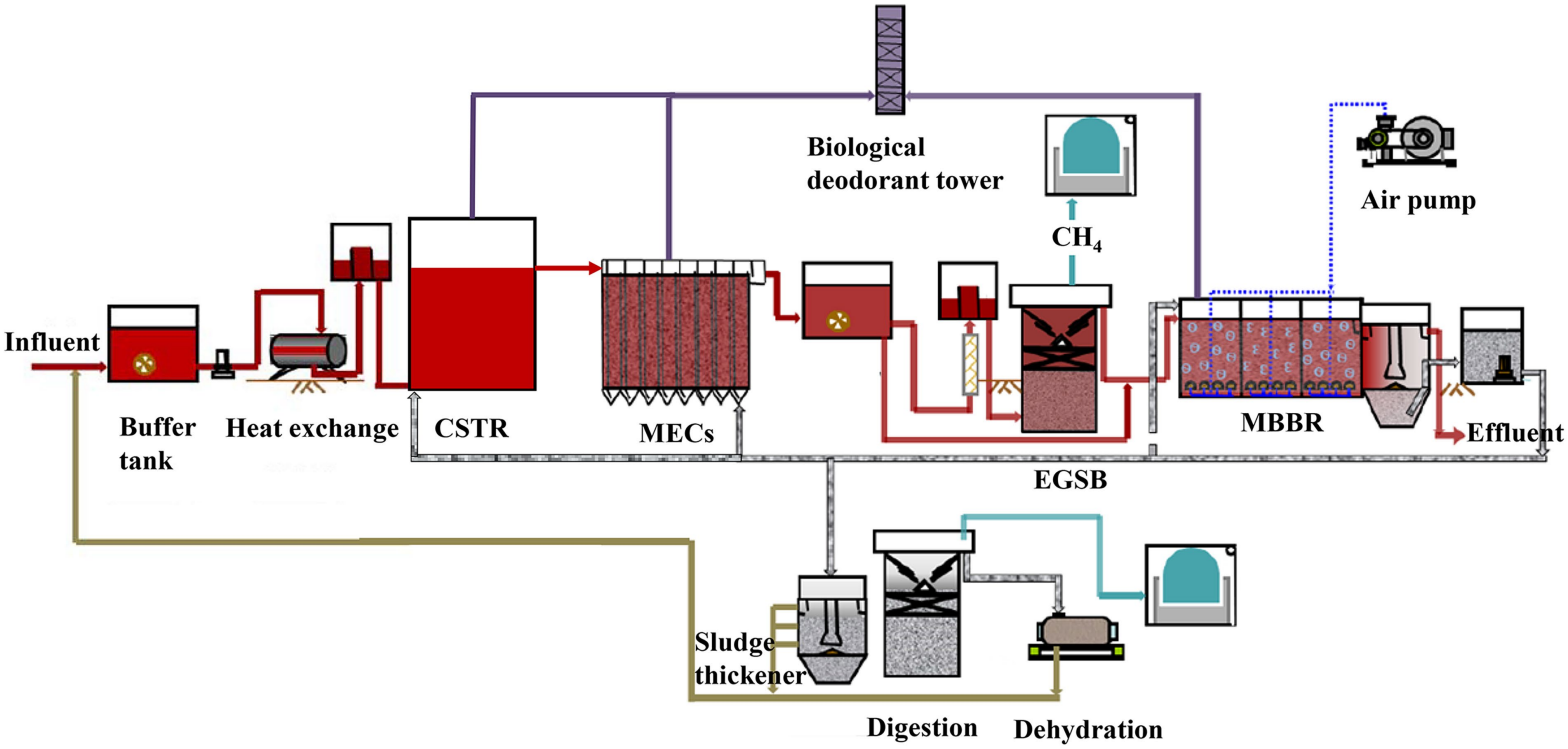
Flowchart of the integrated process of CSTR + MECs + EGSB + MBBR.

This study treated the wastewater of a pharmaceutical factory *via* a pilot-scale plant that used the CSTR + MECs + EGSB + MBBR bacteria enhancement treatment method (Figure 1).

Most of the pharmaceutical sludge and residue are removed from the high-concentration pharmaceutical wastewater in the regulating pool, after which the water temperature is adjusted by the heat exchange tank and the wastewater is lifted to the collection and distribution wells (Figure 1). The pharmaceutical wastewater then enters the CSTR, MECs, EGSB, and MBBR treatment units in turn, where the effluent is separated from the mud and water in the sedimentation tank and the supernatant is discharged after entering the separation tank. The temperatures were maintained through auto-controlled heat exchangers to be 35 ± 2°C for the CSTR, and 20 ± 2°C for the MECs and the EGSB and the MBBR. The CSTR has an approximately 800-liter effective working volume with an internal diameter of 1.1 m and a height of 1.3 m. A continuous and stable automatic operation of the system is achieved *via* the programmable logic controller. This system controls the hydraulic residence time and pH of the CSTR to keep the fluid in the hydrolytic acidification stage. The MECs unit uses enhanced electrochemical microbial degradation of the pharmaceutical wastewater and is composed of a reaction cell, electrode, and power supply system. The MEC unit has a 500-liter effective reaction volume and is divided into eight cells, each of which is baffled up and down. The transformer supplies the applied voltage, and the electrodes are made from carbon fiber. The specific details of the MEC unit can be found in patent CN105347516A. The EGSB unit consists of two column reactors with a diameter of 0.6 m and a height of 2.4 m, with a total effective working volume of about 1,300 L. The MBBR unit consists of two-stage MBBR nitrogen removal equipment, which includes three parts: a micro-oxygen tank, an aerobic tank, and a residual nitrification reflux system (see relevant details in Patent CN105565594A). The effective volume of the aerobic tank is about 300 L with a length of 0.4 m and a width of 0.8 m and a height of 1 m, and the effective volume of the micro-oxygen tank is about 600 L with a length of 0.8 m and a width of 0.8 m and a height of 1 m. The MBBR unit is filled with a multiphase flow dynamic membrane microbial carrier (see Patent CN104787874A). The concentration of dissolved oxygen in the aerobic portion of the MBBR was kept above 2 mg/L. Heterotrophic bacteria in the MMBR further degraded the remaining organic matter in the water to create a favorable autotrophic environment for the removal of nitrogen. The dissolved oxygen was kept below 0.5 mg/L by regulating the micro-oxygen pool through the aeration system. A dissolved oxygen gradient forms inside the biofilm attached to the patent filler, and simultaneous nitrification and denitrification occur inside the reactor. After the hydrolysis and acidification effect of the CSTR stabilized, the influent flow rate was about 1 m^3^/d, and the residence times of the CSTR unit, the MEC unit, the EGSB unit, and the MBBR unit was 19.2, 12, 31.2, and 21.6 h, respectively.

The advantages of the pilot-scale plant in this study lie in its high processing efficiency and low processing cost. At the front end, the refractory organic matter in the wastewater is greatly reduced through the function of the CSTR and the MECs, as is the biological toxicity of the wastewater. At the back end, the efficient granular sludge unit and biofilm unit can be used to strengthen the treatment of organic matter and nitrogen in the wastewater so that the pilot-scale system can complete the efficient removal of the refractory pollutant and nitrogen. Although four reaction units are used in the pilot-scale system studied, they are all carried out by biological or related treating methods, so the cost of treatment is much lower than the operating costs of physical methods such as membrane processing and evaporation. Among them, the cost of the CSTR unit and the MBBR unit is very low due to having a simple operation mode and a mature and stable process, and the main operating costs of the system are to debug and run the MECs unit and the EGSB unit. In addition, in order to facilitate the application and popularization of this technology, we named the system "Integrate Continuous stirred tank reactor, Microbial electroassisted catalysis, Expanded granular sludge blanket and Flow dynamic biofilm (ICMEF)".

For the initial start-up phase, anaerobic granular sludge purchased from the sewage plant was used in the EGSB reactor. However, the remaining sludge from the pharmaceutical factory’s sewage treatment workshop’s secondary sedimentation tank was used to seed the other reactors. The sludge inoculation ratio of each reactor was approximately 30%, and the reactors had an MLVSS/MLSS ratio of 0.75 and an MLVSS of 3,570 mg/L. If the sludge were to be directly injected into the reactor, it would cause serious floating phenomena. Therefore, the sludge needed to be aerated in each reactor for 48 h before sludge inoculation. To improve the bioavailability of wastewater and accelerate the speed of sludge domestication, industrial brown sugar was added to each reactor during aeration. The floating sludge, which was not bioactive in the initial start-up phase, was continuously discharged. After 2 weeks, the sludge has adapted to the anaerobic environment. At this point, the effluent was almost free of any floating sludge. Due to the slow start-up of the anaerobic MECs (Microbial Electrochemical System) and CSTR (Hydrolytic Acidification System) reactors and the poor biodegradability of the pharmaceutical wastewater, brown sugar solution was continuously added during the sludge culture stage of these two reactors. The organic load in the CSTR gradually rose from 1 kg to 8 kg COD/m^3^. Due to significant changes in the water quality, the inlet COD was measured every 12 h. The volume load of the inlet was also calculated to adjust the inlet flow volume. After 6 weeks of operation, the sludge in the system changed from black and odorless to gray and brown. The sludge had no obvious odor, and the effluent was moderately acidic. The COD removal rate remained steady at around 20–30% and was able to withstand significant COD influent fluctuations. At this point, the CSTR reactor has started up successfully. After the CSTR reactor was successfully started, the effluent that entered the MECs started to acclimate to its biofilm. A change in the electrode potential should be detected during the start-up process. The microbial electrical assistance system is considered to have successfully started when the anode potential value is stable. In addition, when the COD removal reaches more than 80% and the operation has been stable for about a week during the EGSB reactor’s starting process, the reactor is successfully started. In the MBBR, the filling ratio of the filler was 40%, while the dissolved oxygen was controlled between 2 and 4 mg/L. Based on the amount of sludge in the two-stage aerobic MBBR tank, the sludge in the sedimentation tank was returned either intermittently or continuously.

### 2.3. Sampling and analysis

Before analyzing the samples, the pooled samples were passed through a 0.45-μm filter. The potassium dichromate method was used to analyze the COD, while NH_4_^+^-N and TN were measured using a spectrophotometer (N6000, Yoke, China). The DO was measured using a DO meter (JPB-607A, REX, China), and the pH was measured with a pH meter (PHB-4, REX, China). The volatile acid content was determined by gas chromatography. For the gas chromatograph (Agilent 7890A, America), the carrier gases were nitrogen and hydrogen, at flow rates of 50 and 55 mL/min, respectively. An FID hydrogen flame detector was used to analyze the samples. An injection volume of 10 L was used at an injection port temperature of 250°C, a column temperature of 240°C, and a furnace temperature of 170°C, which was heated at a 20°C gradient and was maintained for 2 min.

High-performance liquid chromatography on a C18 column (150 × 4.6 mm, 5 μm; Agilent Corporation) was used to detect benzothiazole, indole, pyridine, and quinoline. The mobile phase of the benzothiazole detection was methanol–water (55:45, v/v) at a flow rate of 1 mL/min^−1^. For the benzothiazole detection, the UV light detection wavelength was 280 nm, the injection volume was 10 μL, and the column temperature was 30°C. The mobile phase of the indole was acetonitrile water (50:50, v/v) at a flow rate of 1 mL/min^−1^. For the indole detection, the detection wavelength of the ultraviolet light was 254 nm, the injection volume was 10 μL and the column temperature was 20°C. The mobile phase that was used to detect pyridine was methanol–water (80:20, v/v) at a flow rate of 1 mL min^−1^. The detection wavelength of the ultraviolet light was 254 nm, the injection volume was 10 μL and the column temperature was 20°C. The detection of quinoline was the same as that of pyridine, but at a UV detection wavelength of 275 nm.

## 3. Results and discussion

### 3.1. Removal of conventional metrics

**Figure 2:**
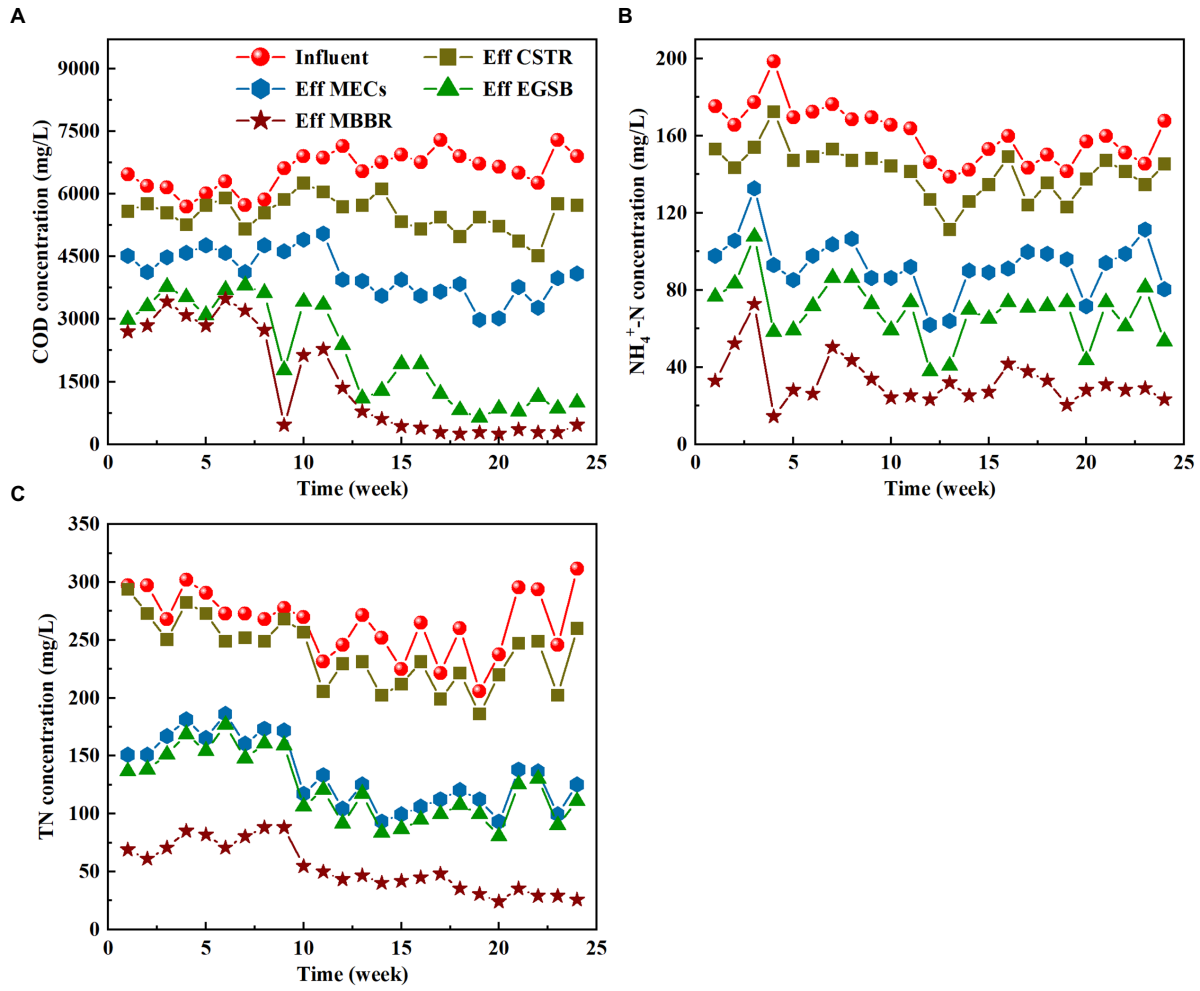
Variation characteristics of COD (**A**), NH4+-N (**B**) and TN (**C**) in different process sections.

The pilot-scale plant achieved stable operation and high removals of dissolved COD (94.83%), NH_4_^+^-N (80.58%), and TN (86.36%) after 13 weeks of domestication (Figure 2; Table 2). Figure 2A shows the COD changes during the start-up and operation. The effluent COD of the CTSR was relatively large in the early stages of operation, when the COD removal rate was only between 4 and 13%. During its initial start-up phase, the CTSR could have lost some of the sludge. The sludge acclimation remained relatively stable throughout week six of the system’s operation, and there was almost no sludge outflow in the effluent. At this time, the COD removal rate gradually increased from 10 to 25%. The increase in the COD removal rate indicates that hydrolyzing acidifying bacteria gradually adapted to their environment, adjusting to the changes in the water quality and initially being able to withstand the impact load. Even though the COD of the influent water varied between 5,500 and 7,800 mg/L from weeks 7 to 24 of the operation, the sludge in the system developed well and changed from a black-odorous sludge to a gray-brown sludge without an obvious odor. The COD removal rate also remained steady throughout at about 25–30%.

**Table 2:** The material balance of each pollutant (COD, NH_4_^+^-N, TN, and persistent pollutants) in the pharmaceutical wastewater treatment flow.

Item	Influent (mg/L)	CSTR effluent (mg/L)	MECs effluent (mg/L)	CSTR+ MECs removal rate (%)	EGSB effluent (mg/L)	MBBR effluent (mg/L)	Total removal rate (%)
COD	6813.74	5323.64	3607.27	47.05	1128.08	352.32	94.83
NH_4_^+^-N	151.96	136.28	92.85	38.9	67.04	29.51	80.58
TN	255.71	220.98	112.39	56.05	100.71	34.88	86.36
Pyridine	28.09	18.54	9.15	67.43	/	5.71	79.69
Benzothiazole	197.61	138.52	10.09	94.89	/	4.62	97.66
Quinoline	44.47	32.67	16.33	63.28	/	8.30	81.34
Indole	33.95	22.16	7.98	76.49	/	1.99	94.13

The average effluent quality after the 13th week.

During the start-up stage of the MECs, the COD removal rate of the effluent of the MECs was between 12 and 28%. The startup of the cathode microorganisms in the MECs was quicker, and the electrode potential of the anode gradually stabilized. After the microbial electric assistance reactor was effectively activated, the electrode potential was stable. The anode potential was stabilized at about −0.35 V, and the cathode potential was stabilized at about −0.9 V. From about the 12th week, the COD effluent was not affected by the inflow fluctuations as much, and the COD removal rate ranged from 30 to 40%. This means that the MEC system removed CODs at a relatively stable rate. The microbial electro-assist system must enrich many electrochemically active bacteria on the electrode surface and ensure their activity. However, many small molecules, such as acetic acid, butyric acid, ethanol, and lactic acid, that are produced during hydrolysis and acidification, are more easily utilized in MECs. This helps the electrochemically active bacteria grow. A large number of microorganisms have survived on the electrode surface of the microbial electrical assistance system after the stable operation of the MECs. Over time, these microorganisms will adapt to the effluent of the CTSR and will catalyze refractory substances to accelerate electron transfer. These microorganisms could deliver tens of thousands of electrons to the cathode, which would continue to transfer to the refractory substances and greatly aid in the degradation of toxic pollutants.

The start-up of EGSB is relatively slow due to the effect of refractory toxic substances such as antibiotics in pharmaceutical wastewater on granular sludge. During the early start-up period of EGSB, a large volume of sludge will flow out of the reactor, including sludge with poor settling performance, dead sludge, and granular sludge that disintegrated during inoculation, while sludge with good settling performance and activity will remain in the reactor. With the improvement of the degradation effect of the front-end CTSR + MECs unit on toxic substances such as antibiotics and the adaptation of the granular sludge in EGSB to antibiotics, the start-up of EGSB was completed after the 13th week. The concentration of COD in the effluent is high during this time. From the 13th week, when the reactor started up, the COD degradation rate dropped to 79.23%, after which it increased slightly during the stable operational stage. The final COD degradation rate maintained a level of about 80% during the stable operational stage of the subsequent reactor. This good degradation rate may be due to the good operating condition of the EGSB and the continuous formation of new antibiotic-resistant granular sludge. The COD removal rate of the biofilm system in the MBBR segment was low, mainly because most of the volatile acids have been consumed by carbon sources in the EGSB stage. The effluent COD fluctuated between 224 and 331 mg/L after finally passing through the sedimentation tank, which indicates that the system still contained some organic matter that was difficult to biodegrade.

The changes in NH_4_^+^-N and TN during the start-up and operational phases are shown in Figures 2B,C. The concentration of NH_4_^+^-N in the influent ranged from 120 to 175 mg/L, while the TN concentration in the influent ranged from 205.13 to 300.58 mg/L. After the CSTR unit treatment, the NH_4_^+^-N concentration increased. This increase in NH_4_^+^-N concentration may be due to the decomposition of some organic nitrogen compounds in the influent water. However, the TN concentration decreased only a little, which may be due to the limited consumption of nitrogen by the proliferation of anaerobic bacteria during the cellular synthesis process. The NH_4_^+^-N and TN concentrations both decreased significantly after passing through the MECs. This may be because ammonia was oxidized to nitrite or nitrate in the air cathode, and then nitrite or nitrate was reduced to nitrogen in the anaerobic environment. The ammonia can be used as an electron donor and reactor between the cathode and anode to provide energy for microorganisms in the bio-electrochemical anammox process in MECs, while the high potential of the anode can further promote nitrification (Liu et al., 2017). The denitrifying bacteria can also use nitrate to degrade organic matter for nitrogen removal because an anaerobic environment is prevalent in the MECs.

The degradation of NH_4_^+^-N and TN occurs when wastewater enters the EGSB, and the efficiency of this degradation is less than that of MECs, which could be due to the elimination of NH_4_^+^-N and TN solely by the process of cellular proliferation in the EGSB. During the start-up stage of the MBBR, the effluent NH_4_^+^-N fluctuates, and the effluent NH_4_^+^-N can be as low as 15 mg/L. The TN is, however, maintained at a high level. During the stable operation stage of the reactor, after about 10 weeks, the total NH_4_^+^-N removal stays stable at about 80%, while the effluent TN continuously decreases. The removal of NH_4_^+^-N mainly depends on the nitrification of nitrifying bacteria. They belong to autotrophic bacteria and have a long generation time. The microbial attachment on the filler surface was unstable because of the toxicity of pharmaceutical wastewater during the early stages of MBBR, slowing the growth and reproduction of nitrifying bacteria. The number of nitrifying bacteria is very low or even nonexistent. With the stable operation of the front-end CTSR + MECs unit and the adaptation of MBBR to wastewater toxicity, the biomass attached to the filler (such as the number of nitrifying bacteria) also gradually increased. The TN in the MBBR can be greatly degraded because the biofilm in the reactor allows the aerobic and anaerobic microenvironments to exist at the same time. This enables simultaneous nitrification and denitrification, which reduces the need for carbon sources in the MBBR (Iannacone et al., 2021).

### 3.2. Variation characteristics of volatile acids

Complex organic matter is anaerobically digested in four stages: hydrolysis acidification, the production of hydrogenation and acetic acid, the production of isoacetic acid, and the production of methane. During hydrolysis acidification, complex organic matter is converted into small molecules such as organic acids and alcohols. Complex organic matter is first broken down to simple organic matter by the extracellular enzymes of anaerobic bacteria. Cellulose is also hydrolyzed into simpler sugars, proteins into simpler amino acids, and lipids into fatty acids and glycerol. Anaerobic fermentation and oxidation under the action of acid-producing bacteria then convert these simple organic compounds into fatty acids such as acetic acid, propionic acid, butyric acid, and alcohols. Anaerobic bacteria and facultative anaerobic bacteria are the main hydrolytic fermentation bacteria involved during this stage (Shi et al., 2017).

**Figure 3:**
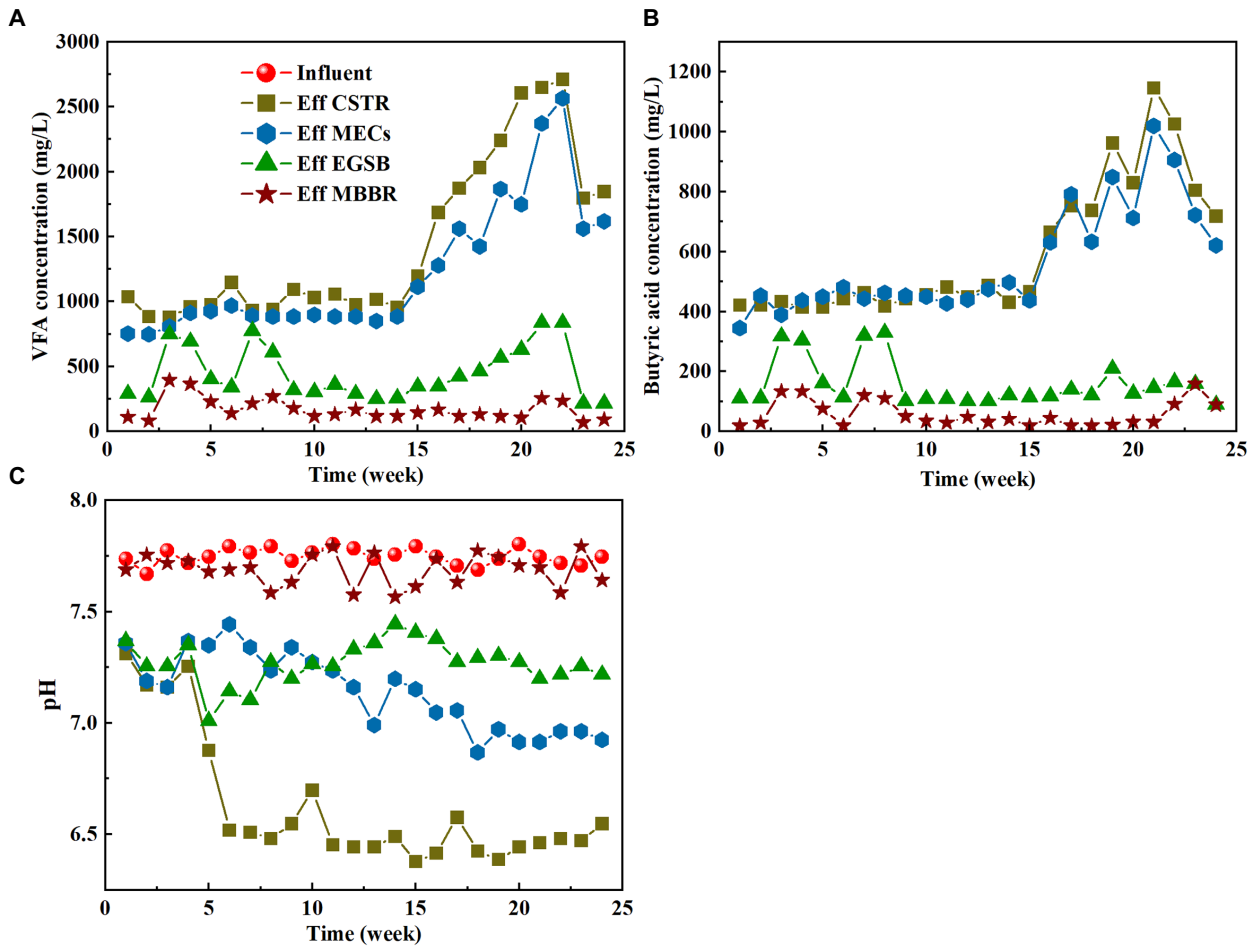
Variation characteristics of VFA (**A**), butyric acid (**B**) and pH (**C**) in different process segments.

The small molecular volatile acids produced by hydrolysis and acidification in the CSTR are substrates that are easily absorbed and metabolized by various microorganisms. Easily degradable organic matter is used for growth by the electrochemical microorganisms in the MECs. These organisms can degrade refractory organic matter through co-metabolism, thereby promoting the removal of refractory organic matter. Improving the yield of volatile acids will greatly assist the MECs in the system. The variations in total volatile fatty acids (VFAs) and butyric acid are shown in Figures 3A,B. The effluent concentration of volatile acid gradually rises as the running time increases in the CSTR and MECs. The wastewater matrix does not have time to acidify during the initial stage of the reaction because of the poor biodegradability and sludge culture, resulting in only a small amount of VFA. The VFA in the hydrolysis section increased sharply after the 14th week. This means that the combination of CSTR and MECs improved the content of the volatile acids to a greater degree by improving the contact between the wastewater and the microorganisms. It also means that the stable habitat in the reactor was more conducive to the growth and multiplication of the hydrolyzing acidifying bacteria. However, the volatile acid of the effluent decreased after 22 weeks. The influent water quality may affect the volatile acid. Figures 3A,B also show that the concentrations of volatile acids and butyric acids in EGSB effluent tend to be stable, indicating that the EGSB anaerobic fermentation process has a stable effect on organic matter degradation. In contrast, volatile acid and butyric acid concentrations in MBBR effluent are extremely low because heterotrophic bacteria could assimilate any easily degradable organic matter.

Volatile acids are generally produced by hydrolytic acidification. The system pH mainly affects the type and acidification rate of the hydrolytic acidification products. Chen et al. (2008) investigated how pH affects the hydrolytic acidification process that is used to treat pharmaceutical wastewater. They found that most of the products were acetic acid and butyrate when the pH was between 4.5 and 6.5 and that the acidification rate increased from 30 to 44% when the pH rose from 5.0 to 5.5. Acid production was suppressed, however, and ethanol was produced as a partial substitute when the pH of the culture was reduced to 4.5. In anaerobic reactors, methanogenesis can only occur under neutral conditions. In contrast to methanogens, hydrolytic acid-producing bacteria are much more pH-tolerant than methanogens. They can still be very active at pH 5 and can even grow up to a pH as low as 3.8 (Li and Tabassum, 2022). Therefore, methanogen growth can be inhibited by keeping the pH of the influent water in the acidic phase. The most suitable pH range for hydrolytic acidification is generally 5.5–6.5, and hydrolytic acidification rates will decrease when the pH increases or decreases above or below this range.

Many organic acids and alcohols are produced during hydrolytic acidification in the CSTR. The accumulation of these substances decreases the pH of the system. The pH of the anaerobic system will have a greater impact on the entire biochemical reaction process compared with an aerobic system. The pH of the inlet and outlet water of the hydrolysis and acidification reactor must therefore be continuously monitored. By measuring the pH change between the inlet and outlet water of the system, the degree of hydrolytic acidification can be determined. Figure 3C depicts the pH changes that occur during the system’s startup and operation. The rate of organic acid production in the hydrolytic acidification reactor was slow because of the low proportion of hydrolytic acidifying bacteria in the start-up phase. Therefore, even though the pH decreased, it only decreased from 7.7 to approximately 7.0 in the first 4 weeks of operation. The hydrolytic pH varied from 6.07 to 6.43 after week 5, and the acidification rate varied from 19.93 to 22.07%, while the effluent pH remained stable. The increased volume load of the inlet water and the increased amount of organic acid produced by the hydrolytic acidifiers influenced these parameters. Although the effluent pH of the CSTR slightly exceeds the optimal pH range of hydrolytic acidification (5.5–6.5), the pH can be controlled by shortening the HRT at a later stage to maintain the optimal hydrolytic acidification effect.

MECs have a small effect on the pH of the system (Figure 3C). The pH of the MEC effluent decreased slightly after week 5, ranging from 6.99 to 7.2 during the start-up stage and between 6.7 and 7.0 during the stable operational stage. Based on the slow and continuous decrease of the pH, it can be deduced that a part of the volatile acid produced by hydrolytic acidification is used by the electrochemical microorganisms in the MECs. The pH of EGSB effluent ranged from 7.03 to 7.28. Because a pH close to neutral promotes the system’s methane production processes, volatile organic acids are primarily consumed during this stage (Figures 3A,B). In the meantime, the NH_4_^+^-N produced by the anaerobic reaction also increases the pH of the EGSB effluent. The pH of MBBR effluent fluctuated within a certain range. The simultaneous removal of carbon and nitrogen by simultaneous nitrification and denitrification processes in MBBR caused this fluctuation.

### 3.3. Removal effect of characteristic pollutants

**Figure 4:**
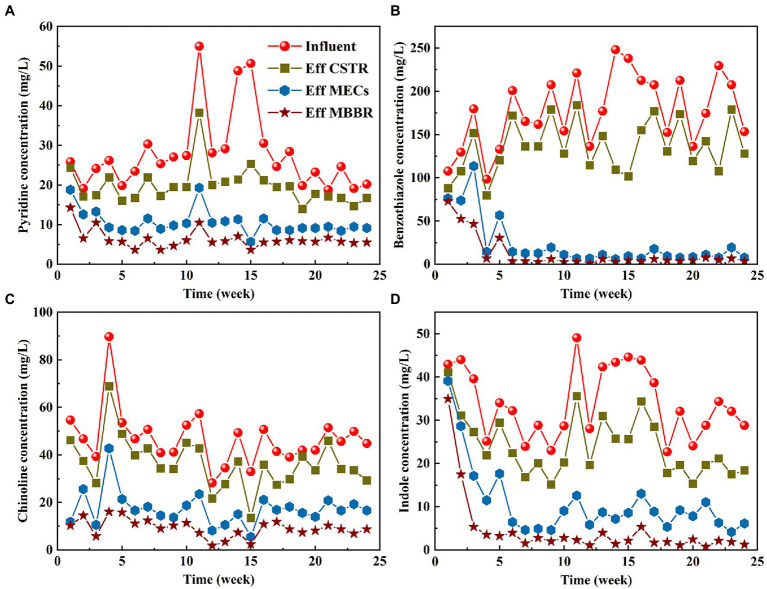
Variation of characteristic pollutants: **(A)** pyridine, **(B)** benzothiazole, **(C)** quinoline, and **(D)** indole.

Four typical toxic pollutants (benzothiazole, pyridine, indole, and quinoline) were selected from pharmaceutical wastewater, and the removal of the four substances was monitored in the start-up and stable operational stages (Figure 4). During the stable operation of the pilot-scale plant, the total removal rates of benzothiazole, indole, pyridine, and quinoline were 97.66, 94.13, 79.69, and 81.34%, respectively (Table 2). The removal rate of five-membered organic compounds (benzothiazole and indole) in this system is higher than that of six-membered organic compounds (quinoline and pyridine). The structure and charge properties of the substances may influence these results. The six-membered heterocycles (quinoline and pyridine) are usually stable, and destroying their ring structure requires more energy. Quinoline and pyridine are also antibiotics that are more toxic than benzothiazole. Pyridine and quinoline had similar chemical structures, both containing a six-membered nitrogen heterocyclic ring, and the key step in their biodegradation was the two initial mono-oxidation reactions leading to ring opening (Tang et al., 2015). The enzymes used to degrade the two pollutants in microorganisms could be similar or the same. The enzymes induced in the degradation process could therefore be used by each other, leading to their competition for intracellular electron donors(2H), and pyridine and quinoline removal rates decelerated during simultaneous biodegradation compared to independent biodegradation (Xu et al., 2017). Previous studies have shown that microorganisms with mono- and dioxygenases, such as Rhodococcus sp., may lead to the addition of hydroxyl in pyridine and quinoline (Martínková et al., 2009; Zhu et al., 2021).

The removal of pharmaceuticals in wastewater treatment plants was mainly achieved through biodegradation, adsorption, chemical oxidation by oxygen, and hydrolysis, of which biodegradation is the most important way (Peng et al., 2019). As shown in Figure 4, in the early stages of reactor start-up, the four characteristic pollutants investigated all had certain removal effects, indicating that biodegradation and sludge adsorption could work in the system. However, each reactor of the combined process had a specific level of effectiveness in removing the four organic pollutants, indicating that biodegradation was the main degradation pathway. The biodegradation of toxic pollutants is usually carried out by matrix co-metabolism. Co-metabolism is defined as “the transformation of a non-growing substrate in the obligate presence of a growing substrate or another transformable compound” (Kora et al., 2020). Compounds that cannot support cell replication due to their low concentration (for example, micropollutants in wastewater) are known as non-growth substrates. A series of biochemical reactions that use active enzymes, is required to remove and degrade the four kinds of toxic pollutants. During co-matrix metabolic degradation, the production of key enzymes is induced during pollutant degradation. In anaerobic or aerobic environments, microorganisms degrade small molecules that are easy to degrade, such as acetic acid. Refractory substances such as benzothiazole, however, approach and induce these enzyme molecules slowly. Refractory substances such as benzothiazole begin to degrade slowly after the induction of enzyme proteins, thereby completing the co-metabolism process. The enzyme that is induced during co-metabolism is non-specific. As a result, it can degrade substances that are easily degradable as well as those that are more difficult to degrade. This results in competition for this enzyme during co-metabolism between substances that are easily degradable and those that are more difficult to degrade. Therefore, the nature and quantity of the growth substrate determine the nature of the co-metabolic process. Kinetic modeling of co-metabolism found that the production of key enzymes during the reaction process can be promoted by increasing the proportion of easily metabolized co-substrate, which improves the degradation of toxic pollutants (Mazioti et al., 2015, 2017). Due to the high concentration of volatile acids in CSTR and MECs (Figure 3A), their co-metabolic ability is stronger than that of EGSB and MBBR, and thus their contribution to toxic pollutant removal is higher. However, there are still some easily degradable organic compounds in EGSB and MBBR, so they can further degrade toxic pollutants. In addition, due to the stable treatment effect of front-end CSTR and MECs units, the concentration of toxic pollutants in EGSB and MBBR decreased significantly (Figure 4), which was conducive to the formation and maintenance of granular sludge and biofilm.

Figure 4 also shows that the MECs contribute significantly to the removal of refractory pollutants. After CSTR and MEC successfully treated the pharmaceutical wastewater, the average removal rates of benzothiazole, indole, pyridine, and quinoline were 94.89, 76.49, 67.43, and 63.28%, respectively (Table 2). This is because, apart from the microbial co-matrix effect that is present in MECs, cathode electrocatalysis also contributes significantly to the reactor’s degradation of toxic pollutants. Systems operating under anaerobic conditions can detoxify nitrophenol wastewater. Nitrophenol wastewater cannot be fully mineralized. The cathode of MECs can provide continuous electrons for refractory pollutants, such as reduced nitrobenzene, decolorization of azo dyes, and reduction of halogenated compounds (Shen et al., 2013). Refractory pollutants such as antibiotics can therefore be partially degraded (nitro reduction and dehalogenation) and/or completely degraded, while their antibacterial activity can also be eliminated. Cathode potential also has an important impact on the removal of pollutants in MECs. Kong et al. (2015) explored the influence of different cathode potentials (−0.15 to −1.25 V) on the degradation rate constant (k) and removal efficiency of antibiotics. They found that it increased significantly with a decrease in the cathode potential. However, Guo et al. (2018) studied the effect of the cathode potentials (−1.25, −1, and − 0.5 V) on the removal of antibiotics and resistance genes and found that antibiotics were most effectively removed at −1 V and that the resistance genes were lower. The results of this study were similar to the cathode potential results of our study. The cathode potential in our study was controlled at −0.9 V.

### 3.4. Analysis of degradation pathways of characteristic pollutants

In our study, ion chromatography was used to analyze the inorganic products in the effluent, while a gas chromatograph-mass spectrometer (GC–MS) was used to test mixed samples of effluent that had been aerated for two, four, and 7 h. The parameters, such as retention time and the relative molecular weight of the main intermediates of the benzothiazole degradation process, were obtained from the GC–MS analysis (Table 3).

**Table 3:** List of degradation pathway compounds.

Chemical structure	Chemical formula	Formula weight (g/moL)	Retention time (min)
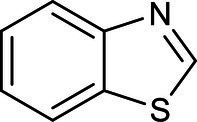	C_7_H_5_NS	135	8.953
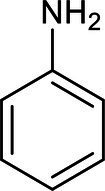	C_6_H_7_N	93	2.938
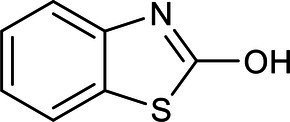	C_7_H_5_NOS	151	4.045
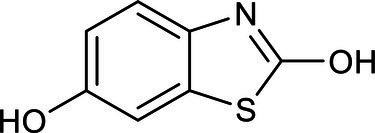	C_7_H_5_NO_2_S	167	8.012
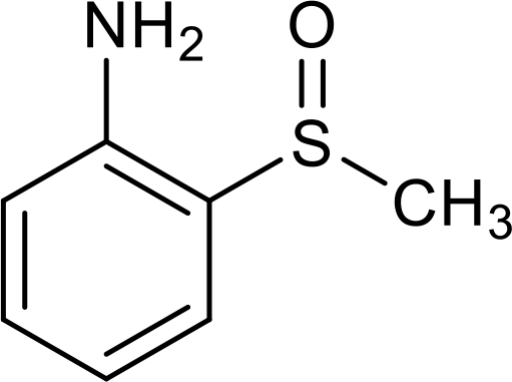	C_7_H_9_NOS	155	12.315
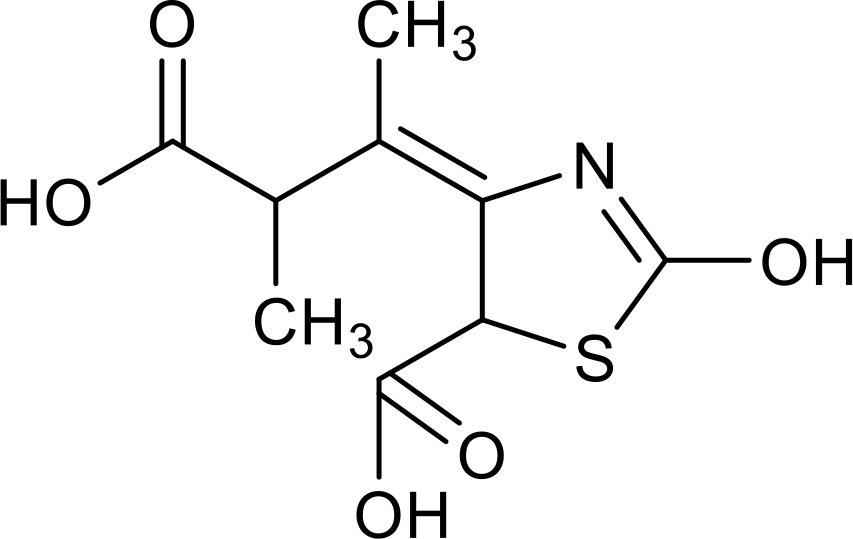	C_9_H_9_NO_5_S	203	16.347
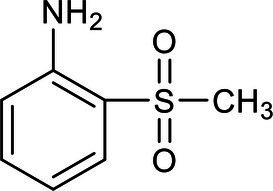	C_7_H_9_NO_2_S	171	15.236

**Figure 5:**
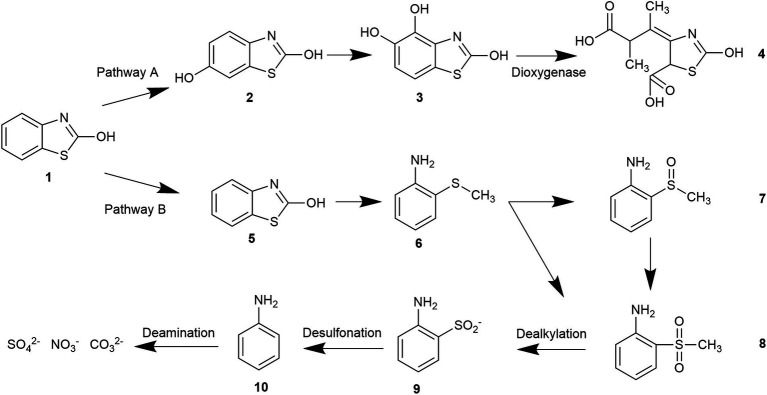
Two degradation pathway of 2-hydroxybenzothiazole.

The degradation pathway of benzothiazole was inferred from the results of the intermediate products and the ion chromatography. The degradation of benzothiazole typically begins with the hydroxylation of the heterocycle and proceeds with the cleavage of the ring since it is a heterocyclic compound. The five-membered ring adjacent to the six-membered ring usually was oxidized first to form 2-hydroxybenzothiazole (OBT) during benzothiazole degradation. The mass concentrations of benzothiazole and OBT in the MEC reactor were determined by high-performance liquid chromatography. It was found that OBT concentration increased with the decrease in benzothiazole concentration. When benzothiazole concentrations were low, OBT concentrations reached a peak and then began to decline. Therefore, OBT was considered to be the intermediate product of benzothiazole. According to the literature research, we could find that OBT may have two biodegradation pathways (Haroune et al., 2002; Liao et al., 2018). Further, based on the results of intermediates analyzed by GC–MS and ion chromatography, two specific degradation paths for OBT degradation were deduced (Figure 5).

Pathway A: OBT (1) undergoes hydroxylation and further degrades to form 2,6-dihydroxybenzothiazole (2) under the action of microorganisms. Benzothiazole-2,4,5-triol (3) was mainly formed during the further hydroxylation of 2,6-dihydroxybenzothiazole (2), due to the different activities at the different benzene ring substitution sites. Under the continuous action of the microorganism’s dioxygenase, the final benzene ring underwent a ring-opening reaction, which caused the formation of the acidic substance (4).

Pathway B: OBT first generates 2-sulfobenzothiazole (5), and S and N heterocycles are cleaved by microorganisms. A desulfonate group reaction takes place during this process, and part of the organic sulfur is converted to inorganic sulfate to form 2-(methylthio)aniline (6). This desulfonic acid group reaction is very important for removing the toxic and irritating odor of benzothiazoles and their derivatives. Further oxidation converts the 2-(methylthio)aniline (6) to 2-(methylsulfinyl)aniline (7) and 2-(methylsulfonyl)aniline (8). The continuous action of the microorganisms further converted the resulting 2-(methylsulfinyl)aniline (7) to 2-(methylsulfonyl)aniline (8). Microorganism-produced enzymes further decompose or hydrolyze the 2-(methylsulfonyl)aniline (8), resulting in dealkylation and the formation of the 2-aminobenzenesulfinate (9). The 2-aminobenzenesulfinate (9) was further desulfated and deaminated, and it was finally mineralized to CO_3_^2−^, NO_3_^−^, and SO_4_^2−^.

When comparing and analyzing the degradation pathways of these two benzothiazoles, it can be seen that the main intermediate metabolite of pathway A is an acidic substance (4). Pathway A is the ring opening reaction of the benzene ring. However, the main metabolites of pathway B are 2-(methylsulfinyl)aniline (7) and 2-(methylsulfonyl)aniline (8). Pathway B is ring-opening reactions of heterocycles. The complete mineralization of benzothiazole requires the cleavage of the heterocycles.

Ion chromatography was used to detect the inorganic products of the effluent. The concentration of SO_4_^2−^ was determined to be 15 mg/L. Nessler’s reagent spectrophotometry was used to detect the NH_4_^+^-N concentration in the inlet and outlet water. In the inlet water, the NH_4_^+^-N concentration was not detected or was below the detection limit, while the NH_4_^+^-N concentration in the outlet water was 11.533 mg/L. This means that the NH_4_^+^ and SO_4_^2−^ were produced during the reaction. Pathway B is therefore considered to be the main pathway for the degradation of benzothiazole. Acidic substance (4) was also detected by GC–MS, but at low concentrations, and therefore path A is the auxiliary path of the benzothiazole degradation.

## 4. Conclusion

This study used the combined CSTR + MECs + EGSB + MBBR to treat actual pharmaceutical wastewater. This system proved to be effective in removing pollutants from pharmaceutical wastewater. We also analyzed the degradation of the main characteristic pollutants in pharmaceutical wastewater in detail. The hydrolytic acidification unit, when used as a pre-treatment unit, can adapt well to the refractory substances contained in the wastewater and has a certain tolerance for toxic substances. Compared with the hydrolytic acidification unit, the bioelectrochemical system used in this study provides a greater contribution to the removal of organic pollutants and can also partially remove conventional pollutants. The EGSB + MBBR unit, which is the main biochemical process at the back end, removed most of the nutrients and further reduced characteristic pollutants. This study provides experimental data and feasible suggestions for the design and modification of a full-size PWWTP.

## Data availability statement

The original contributions presented in the study are included in the article/supplementary material, further inquiries can be directed to the corresponding authors.

## Author contributions

WD: conceptualization, methodology, supervision, writing original draft. J-WP, JD, Y-QW, L-YZ, N-QR, and S-SY: data collection and analysis, conceptualization, supervision, validation, writing and editing. All authors contributed to the article and approved the submitted version.

## Funding

This work was supported by the National Natural Science Foundation of China (Grant No. 52170073); the National Engineering Research Center for Bioenergy (Harbin Institute of Technology, Grant No. 2021A001), and the Open Project of State Key Laboratory of Urban Water Resource and Environment (Harbin Institute of Technology) (Grant No. 2022TS35).

## Conflict of interest

J-WP was employed by China Energy Conservation and Environmental Protection Group, CECEP Talroad Technology Co., Ltd.

The remaining authors declare that the research was conducted in the absence of any commercial or financial relationships that could be construed as a potential conflict of interest.

## Publisher’s note

All claims expressed in this article are solely those of the authors and do not necessarily represent those of their affiliated organizations, or those of the publisher, the editors and the reviewers. Any product that may be evaluated in this article, or claim that may be made by its manufacturer, is not guaranteed or endorsed by the publisher.

## Abbreviations

PWWTP: Pharmaceutical wastewater treatment plants
CSTR: Continuous stirred tank reactor
BES: Bioelectrochemical systems
MFCs: Microbial fuel cells
MECs: Microbial electrolysis cells
EGSB: Expanded granular sludge blanket
MBBR: Moving bed biofilm reactor
COD: Chemical oxygen demand
NH_4_^+^-N: ammonia nitrogen
TN: Total nitrogen
VFA: Volatile fatty acid
GC–MS: gas chromatograph-mass spectrometer
OBT: 2-Hydroxybenzothiazole
